# The Dysexecutive Syndrome Associated with Ischaemic Vascular Disease and Related Subcortical Neuropathology: A Boston Process Approach

**DOI:** 10.3233/BEN-2009-0237

**Published:** 2010-06-10

**Authors:** Melissa Lamar, Cate C. Price, Tania Giovannetti, Rod Swenson, David J. Libon

**Affiliations:** ^1^Department of PsychologySection of Brain MaturationInstitute of PsychiatryKing’s College LondonLondonUK; ^2^Department of Clinical and Health PsychologyUniversity of FloridaGainesvilleFLUSA; ^3^Department of PsychologyTemple UniversityPhiladelphiaPAUSA; ^4^Department of NeuroscienceUniversity of North Dakota Medical SchoolFargoNDUSA; ^5^Department of NeurologyDrexel UniversityPhiladelphiaPAUSA

**Keywords:** Ischaemic vascular disease, white matter, dementia, aging, executive functioning

## Abstract

The introduction of diagnostic criteria for vascular dementia has helped to re-define the impact of various subcortical neuropathologies on aging; however, state-of-the-art neuroimaging techniques and autopsy studies suggest that not all structural brain alterations associated with vascular dementia are exclusive to this neurodegenerative process alone. Thus, a detailed analysis of the cognitive phenotype associated with ischaemic vascular disease is key to our understanding of subcortical neuropathology and its associated behaviors. Over the past twenty years, we have operationally defined this cognitive phenotype using the Boston Process Approach to neuropsychological assessment. This has led to both an empirical, as well as a theoretical understanding of three core constructs related to the dysexecutive syndrome associated with ischaemic vascular disease affecting periventricular and deep white matter as well as subcortical structures connecting these regions with the prefrontal cortex. Thus, difficulties with mental set, cognitive control and mental manipulation negatively impact executive functioning. This review will outline the subtle markers underlying this prefrontal dysfunction, i.e., the dysexecutive phenotype, associated with ischaemic vascular disease and relate it to fundamental impairments of gating subserved by basal ganglia-thalamic pathways within and across various dementia syndromes.

## 1. Introduction

The modern history of vascular dementia dates from the seminal work of Hachinski [1] who introduced the concept of multi-infarct dementia (MID) to explain the interrelationship between cerebral atherosclerosis and cognitive decline often seen in older adults [1,2]. The concept of MID quickly became synonymous with virtually all vascular dementia syndromes regardless of aetiology. The introduction of diagnostic criteria for vascular dementia from the Alzheimer's Disease Diagnostic and Treatment Centers (ADDTC) [3] and the National Institute of Neurological and Communicative Disorders and Stroke – Association Internationale pour la Recherché et l'Enseignement en Neurosciences (NINDS-AIREN criteria) [4] have helped to re-define the impact of ischaemic vascular disease characterized

ISSN 0953-4180/09,10/$17.50 © 2009/2010 – IOS Press and the authors. All rights reserved by lacunar infarcts and white matter alterations seen as white matter hyperintensities or areas of high signal on T2-weighted MRI in pathological aging [5]. Furthermore, the acknowledgement of the role vascular risk factors such as hypertension and diabetes play in brain structure and function [6–8] and the development of vascular cognitive impairment [9,10] belies the increasing impact of ischaemic vascular disease on normal aging, risk for and development of dementia.

Despite these advances, state-of-the-art neuroimaging techniques and autopsy studies suggest that not all structural brain alterations associated with ischaemic vascular disease fit within a common diagnostic rubric of dementia. In fact, among some patients, there may be overlap with respect to the neuropathology underlying the clinical presentation of their specific neurodegenerative syndrome [11–13]. For example, over 30% of patients diagnosed with vascular dementia (VaD) who exhibit periventricular and deep white matter alterations as well as lacunar infarctions have concomitant Alzheimer's disease (AD) (see [14] for review) while upwards of 40% of pathologically confirmed AD cases also present with significant periventricular and deep white matter alterations [15,16]. White matter alterations and strategic involvement of subcortical structures (e.g., the basal ganglia) may also alter the dementia profile of other age-related neurodegenerative processes including Parkinson's disease. In fact, accumulating evidence suggests that ischaemic vascular disease characterized by white matter alterations and lacunar infarctions influences the symptoms, signs and course of a dementia irrespective of dementia subtype [12,17–20].

Over the past twenty years, we have documented the impact of ischaemic vascular disease, i.e., periventricular and deep white matter alterations as well as lacunar infarctions located in the centrum semiovale and/or various nuclei within the basal ganglia, thalamus and pons, on neuropsychological functioning in patients with dementia using the Boston Process Approach to neuropsychological assessment in order to provide a finer analysis of behavioral performance in dementia [21]. Our initial worked focused on distinctions between patients diagnosed clinically with VaD using the ADDTC criteria and patients diagnosed with AD using the NINDS-ADRDA criteria. Given that our reliance on clinical diagnoses could be influencing our conclusions related to ischaemic vascular disease we also investigated the presence of white matter hyperintensities and lacunar infarctions detectable on T2-weighted MRI regardless of dementia diagnosis and further augmented these studies with work directly assessing dementia in other 'subcortical' populations and the role of vascular risk factors on the cognitive phenotypes of dementia diagnoses like AD. Taken together, this work has led to both an empirical, as well as a theoretical understanding of three core constructs related to a distinct dysexecutive syndrome associated with brain alterations within periventricular and deep white matter as well as within subcortical structures with afferent and efferent connections to the prefrontal lobes.

The core constructs include pathological inertia, bradyphrenia and disengagement and temporal reordering deficits. Pathological inertia suggests the continual repetition of an ineffective response style either through the inability to terminate an appropriate behavior once initiated and/or the inability to inhibit an irrelevant response. Bradyphrenia, traditionally associated with 'cognitive slowing' or long response latencies [22] also encompasses a more pervasive alteration of cognitive processing that separates the temporal progression of knowing *what to do* from knowing *how to do it* and leads to a state of fleeting behavior control. Lastly, deficits in disengagement and temporal re-ordering manifest when multiple cognitive operations must be mentally managed and manipulated before a response is produced. Thus, we will provide a detailed analysis of our work outlining the dysexecutive phenotype associated with ischaemic vascular disease characterized by periventricular and deep white matter alterations and/or lacunar infarctions located in the centrum semiovale and/or various nuclei within the basal ganglia, thalamus and pons both related to and regardless of dementia diagnosis. Through this review we hope to demonstrate that the dysexecutive phenotype associated this form of neuropathology is related to fundamental impairments of gating subserved by basal ganglia-thalamic pathways within and across various dementia syndromes.

## 2. The dysexecutive phenotype

### *2.1. Maintaining mental set and pathological inertia*

Similar to ideas put forth by other researchers (i.e. Dias et al. [23] and Rogers et al. [24]), we define the ability to establish and maintain a mental set as the ability to appreciate and understand the nature of a task and to respond within the context of that task until the task is completed. When the ability to maintain a proper mental set is impaired, errors in performance may manifest as perseverations such as being 'stuck in set' [25], or a complete inability to engage at the level of task demands. When examined in detail the presence of ischaemic vascular disease, or vascular risk factors known to contribute to ischaemic vascular disease [7, 17], can significantly impact these errors of mental set.

We have shown that patients diagnosed with ADDTC confirmed VaD (criteria which includes brain imaging but does not delineate specific distributions or severity of the neuropathology) produce significantly more perseverative errors than patients diagnosed with NINDS-ADRDA confirmed AD (criteria that do not explicitly mention the presence or absence of ischaemic vascular disease). Furthermore, the perseverations produced by patients with VaD are distinctly different as compared to the perseverations produced by their AD counterparts [26]. This was illustrated by examining the perseverations produced by dementia patients on the Graphical Sequence Test – Dementia Version (GST-D, [26, 27], a task based on the observations of A.R. Luria [28], designed to induce various types of motor perseverative behavior (Fig. 1).

Patients diagnosed with VaD disproportionately generate *hyperkinetic/interminable* perseverative errors indicative of persistent motor responses while patients with AD display conceptual confusion over distinct forms of motor output resulting in *semantically*-based perseverations. Thus, after the command series "Draw a circle, draw a circle, draw a circle . . ." individuals with VaD continue to produce circles beyond the number required even though the examiner had ceased to ask that more be drawn (Fig. 1, top panel). This pattern of VaD performance, pervasive across the entire GST-D, correlated with poorer performance on tests of executive and motor functioning. In contrast, when asked to *write* the sentence "three squares and two circles," AD patients might *draw* three squares and two circles; or when asked to *draw* a house to the bottom of a flower, AD patients often *wrote* the word 'house' next to the picture of a flower or vice versa (Fig. 1, bottom panel). This AD pattern of performance correlated with tests of naming and semantic knowledge. It appears that the ischaemic vascular disease associated with our patients diagnosed with VaD by ADDTC criteria (determined post-hoc to include lacunar infarctions primarily located in the centrum semiovale and/or various nuclei within the basal ganglia, thalamus and pons in addition periventricular and deep white matter alterations) promotes a more severe impairment of the conscious termination of a simple motor behavior when compared to the more conceptual mistakes produced in the presence of relatively less MRI detectable white matter alterations but significant AD-related neuropathology.

When using a qualitative error analysis based on the Boston Process Approach to neuropsychological assessment, higher-level conceptual versus lower-level pervasive difficulties in establishing and maintaining mental set are also noted on a test of verbal concept formation. Briefly, we re-coded incorrect, i.e., zero-point, responses produced on the WAIS-R Similarities subtest [29] to reflect patients' ability to attain mental set even at a superordinate level. Individuals with ADDTC confirmed VaD produced incorrect responses that were blatantly *out of* set (Q: How are a dog and a lion alike? A: *one barks and the other growls*); errors associated with other executive deficits. By contrast, patients with AD produced zero-point errors that were vague, but nonetheless superficially related to the given word pair (Q: dog-lion; A: *they're alive*). The errors produced by patients with AD were associated with poor performance on measures of language and semantic knowledge [30]. This study combined with our work in motor perseverative behavior suggests that underlying ischaemic vascular disease like that associated with VaD promotes a *dysexecutive* profile characterized by a pervasive impairment in the engagement and/or termination of behavior in compliance with task demands.

Not only does ischaemic vascular disease like that seen in our patients with VaD promote a more severe form of dysexecutive deficits regarding patients' ability to establish and maintain a proper mental set, but vascular risk factors like hypertension and diabetes that promote various forms of ischaemic vascular disease including periventricular and deep white matter alterations as well as lacunar infarctions [6,31] also result in more severe dysexecutive behavior in dementia. African Americans with AD and co-morbid hypertension show significantly poorer performance on executive indices from the Mattis Dementia Rating Scale reflecting initiation and perseveration as well as abstract concept formation when compared to normotensive African Americans with AD [32]. Regardless of race, increasing numbers of vascular risk factors combined with mild to moderate AD is associated with decreasing verbal reasoning and set shifting skills [33]. Upon closer inspection, increasing numbers of vascular risk factors in patients with AD was associated with increasing numbers of *out of* set zero-point error responses on the WAIS-III Similarities subtest [34,35]; a pattern of impairment previously associated with ischaemic vascular disease in VaD [30]. This work suggests that vas-

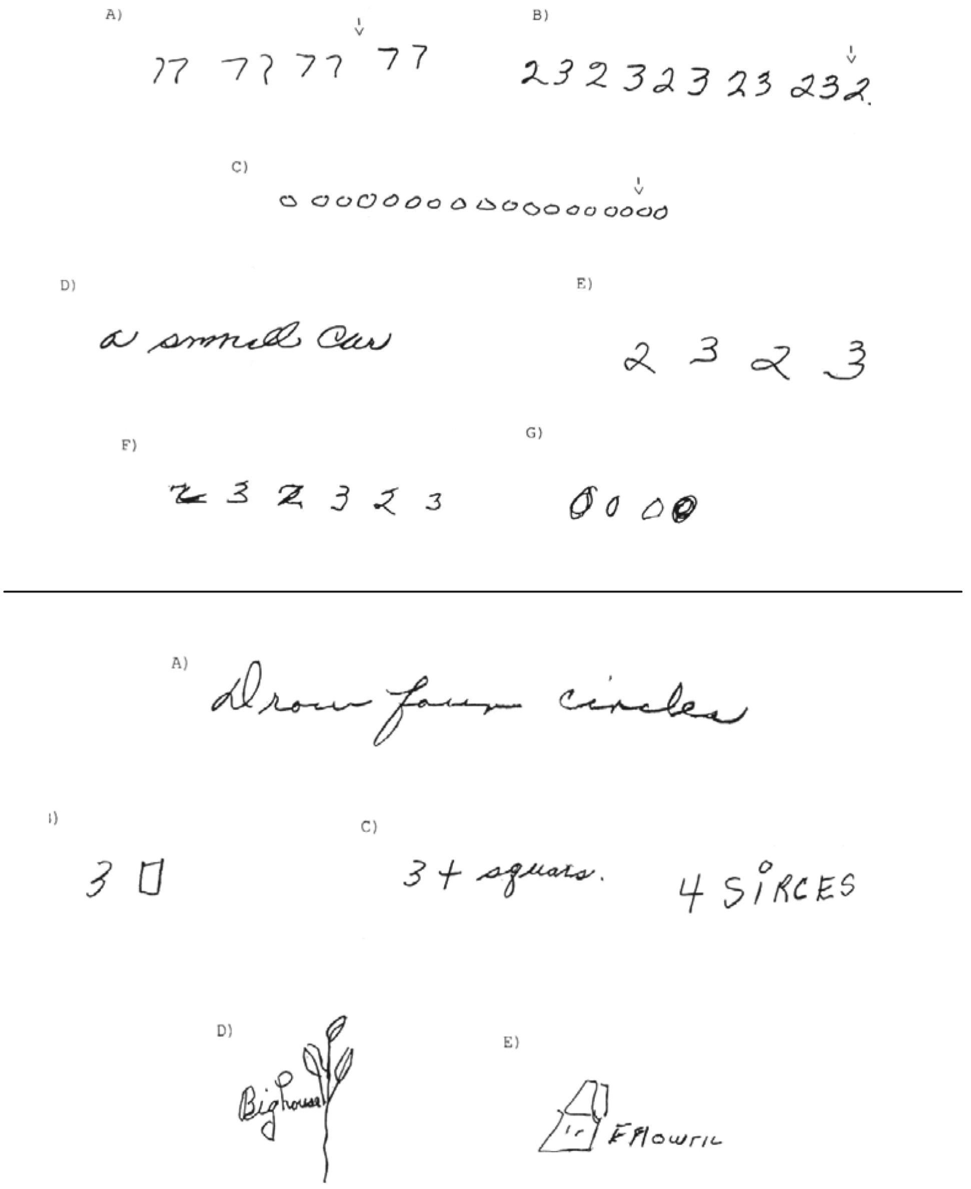

Fig. 1. Top panel display various hyperkinetic perseverations (A-G) made by individuals with vascular dementia associated with white matter alterations. Bottom panel displays various semantic perseverations (A-E) made by individuals with Alzheimer's disease and little to no white matter alterations.

cular risk factors promoting ischaemic vascular disease including periventricular and deep white matter alterations [36,37] may subtly alter the cognitive phenotype of AD toward a dysexecutive profile akin to VaD. While additional work needs to be done to verify that the ischaemic vascular disease associated with vascular risk factors in our patients with AD negatively impacts similar white matter regions as those seen in our patients with VaD, it appears that our studies across dementia subtypes promote the impact of ischaemic vascular disease on a specific form of executive dysfunction within mental set regardless of dementia diagnosis.

The unique pattern of performance seen in our mental set research suggests an underlying pathological inertia like that seen following prefrontal lesions extending into associated subcortical structures of the basal ganglia [28]. Regardless of whether the patient is asked to respond motorically or verbally, the inability to establish and/or maintain the proper mental set under the simplest of conditions (e.g., ‘draw a circle’ or ‘how are a fork and a spoon alike?’) suggests continual repetition of an ineffective response style. Such repetition is typically seen following prefrontal lesions that involve the structural or functional integrity of the basal ganglia [28]; across our studies, however, such repetition or pathological inertia is seen in conjunction with ischaemic vascular disease that directly or indirectly [via neural (dis-)connectivity] impacts the basal ganglia. Thus, the presence of ischaemic vascular disease in VaD or the presence of vascular risk factors that appear to promote a similar form of ischaemic vascular disease in AD, result in mechanistic or lower level processing that prohibits behavior, once initiated, from being appropriately terminated and prevents the inhibition of irrelevant responses. These behaviors ultimately contribute to a core deficit involving pathological inertia. It is as if there is a decoupling between the production of a response and the monitoring or gating of that response during and after task completion that may be related to a common underlying neuropathology.

### 2.2. Cognitive control and bradyphrenia

Cognitive control requires aspects of interference inhibition, flexibility of response selection, as well as sustained attention. When impaired, patients may be unable to inhibit irrelevant responses and produce errors of commission, overlook appropriate responses in the form of omission errors, or become unable to sustain performance across a specific period of time. When examined in detail, the presence of ischaemic vascular disease and related subcortical involvement across dementia subtypes appears to selectively impact the production of these errors as well as the natural progression of behavioral performance over a specified period of time.

Errors of commission and omission appear to be differentially affected by disorders involving ischaemic vascular disease. This was observed in a study examining performance on simple and more complex mental control tasks derived, in part, from the Wechsler Memory Scale (WMS) Mental Control subtest [38]. Patients with ADDTC confirmed VaD – found post-diagnosis to have lacunar infarctions primarily located in the centrum semiovale and/or various nuclei within the basal ganglia, thalamus and pons in addition to their periventricular and deep white matter alterations – not only made more total errors than their AD counterparts regardless of the complexity of the task, the greatest proportion of errors produced in VaD were errors of commission [39]. The pattern of impairment in VaD was most evident at the beginning and end of each mental control task whereas errors in AD were equally distributed across the self-paced duration of task completion [39]. This suggests that the ischaemic vascular disease underlying VaD may impede the ability to select appropriate targets and more importantly, to inhibit erroneous ones suggesting a more severe executive deficit.

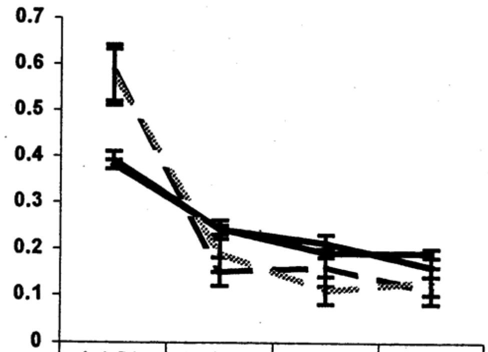

| | 1st Qtr | 2nd Qtr | 3rd Qtr | 4th Qtr |
|---|---|---|---|---|
| NC | 0.38 | 0.24 | 0.19 | 0.19 |
| AD | 0.39 | 0.24 | 0.21 | 0.16 |
| IVD | 0.58 | 0.15 | 0.16 | 0.11 |
| PD | 0.57 | 0.19 | 0.11 | 0.13 |

Fig. 2. The proportion of letter ‘S’ output per 15 second epoch by subject group (NC = normal control; AD = Alzheimer’s disease; IVD; = ischaemic vascular dementia; PD = Parkinson’s disease with dementia).

In order to test patterns of cognitive control across other forms of dementia involving white matter abnormalities and related subcortical neuropathology, we detailed response production across the four 15-second quadrants from tests of letter fluency (letters ‘FAS’). When controlled for total output, dementia patients with ischaemic vascular disease associated with VaD and those with neuropathology involving white matter regions and nuclei of the basal ganglia associated with dementia in Parkinson’s disease (dPD) generated a larger percentage of words during the first 15-seconds when compared to individuals with comparatively less ischaemic vascular disease and/or basal ganglia involvement including patients with AD and healthy controls [39] Fig. 2. However, after the first 15-second epoch dPD and VaD output dropped precipitously below that seen in AD and healthy controls. In contrast, patients with AD were no different from healthy controls in terms of the percentage of output for the remainder of the task. Much like the performance of individuals with VaD and ischaemic vascular disease found to involve the basal ganglia in addition to periventricular and deep white matter, patients with dPD and neuropathology negatively impacting the basal ganglia were unable to maintain a consistent level of performance throughout the length of the letter fluency task.

Both the production and temporal nature of the cognitive control deficits associated with ischaemic vascular disease and/or subcortical (predominantly) basal ganglia involvement suggests an inability to actively engage inhibitory mechanisms as well as a more overarching difficulty initiating and maintaining cognitive control for the duration of performance. Thus, the fact that the majority of errors were errors of commission suggests an inability to accurately monitor and inhibit irrelevant responses; that these errors occurred at particular points of the task coupled with the fact that letter fluency production peaked within the first 15 seconds of the task, suggests that differential impairment in cognitive control in ischaemic vascular disease is not due to response latency but differences in response styles. Not only were individuals with VaD and ischaemic vascular disease found to involve the basal ganglia in addition to periventricular and deep white matter unable to maintain adequate performance for more than a short period of time, this response style was also seen in individuals with dPD, a neurodegenerative disease also associated with basal ganglia involvement. Thus, individuals with common ischaemic vascular disease and/or basal ganglia involvement appear unable to sustain the necessary cognitive control over an extended period of time.

It is possible that a type of *mental* bradyphrenia – not a cognitive slowing or lengthened response latency per se – but something akin to deficits in motor initiation and execution where knowing *what to do* was impeded by an inability of knowing *how to do it* is at work during cognition in the presence of ischaemic vascular disease associated with VaD and dPD. In conjunction with increased commission errors secondary to an inability to adequately engage inhibitory mechanisms, this *mental* bradyphrenia or transient state of cognitive and temporal control of behavior contributes to the dysexecutive syndrome of cognitive control seen in ischaemic vascular disease and related subcortical (i.e., basal ganglia) neuropathology.

### *2.3. Mental manipulation and disengagement and temporal re-ordering*

Working memory is a key component of executive functioning much like encoding is a key component of memory. Baddeley's model of working memory [40] proposes that the brain has lower level, modality specific buffer systems that store and rehearse information and a higher-level central executive responsible both for overseeing these buffer systems and executing processes like the manipulation of information [41]. We have found key aspects of Baddeley's model are particularly impaired in the presence of ischaemic vascular disease. These key aspects center on higher-level processes of disengagement and temporal re-ordering recruited during the mental manipulation of retrieved information when multiple mental operations must be managed and executed before a response is produced [42].

We designed a Backward Digit Task (BDT) [43] similar to the WAIS-III Digit Span Backward subtest to tease apart specific components of Baddeley's model of working memory and implemented it across various dementia subtypes including VaD, AD and mixed dementia to gain a better understanding of the role of ischaemic vascular disease and related subcortical neuropathology in pathological aging. Participants were required to repeat 3, 4, and 5-span trials in reverse order; however, the variables of interest distinguished lower-level short-term storage and rehearsal deficits by quantifying the number of digits recalled in ANY order from higher-level disengagement and temporal re-ordering deficits by quantifying the number of digits recalled in correct SERIAL order. Interestingly, lower-level 'ANY order' skills involving short-term storage and rehearsal were equivalent across all dementia participants regardless of the severity of ischaemic vascular disease as measured by a 40-point visual rating scale of periventricular and deep white matter hyperintensities as seen on T2-weighted MRI. In contrast, patients with dementia associated with at least a moderate degree of white matter damage (i.e., an average of 18 points on our visual rating scale) displayed significant deficits in higher-level disengagement and temporal re-ordering [43]. A closer inspection of these 'SERIAL order' errors revealed that dementia patients with at least a moderate degree of white matter damage showed impaired working memory, due in part, to an inability to disengage from or inhibit over-learned and automatic response tendencies [26,44].

We devised a visual analogue to the verbally mediated BDT called the Object Span Test (OST) where patients are shown a series of 2, 3, and 4 simple two-dimensional shapes and asked to draw the objects in the order they were presented. OST performance in patients with dementia (i.e., VaD, AD and mixed) and at least a moderate degree of white matter damage was consistent with the disengagement and temporal re-ordering deficits seen in a similar dementia sample performing the BDT [45]. Taken together, the pattern of performance seen across the BDT and OST suggest a pervasive impairment of verbal as well as visual working memory abilities in the presence of at least a moderate degree of periventricular and deep white matter damage regardless of dementia subtype due, in part, to the inability to inhibit automatic or irrelevant responses [44]. By limiting our working memory tasks to two to five items, we capitalized on the ability of our participants to perform lower-level storage and rehearsal while taxing them on higher-level disengagement and temporal re-ordering skills.

It is primarily these higher-level disengagement and temporal re-ordering skills that are thought to be heavily dependent not only on the normal functioning of Baddeley's central executive [46] but also the normal functioning of white matter connections associated with the dorsolateral prefrontal cortex [47–49]. In addition to the prefrontal cortex and associated white matter tracts, structures of the basal ganglia including the caudate and the dorsomedial nucleus of the thalamus participate in the networks of cortical-striato-thalamo-cortical loops that interact with the dorsolateral frontal lobe. When the core constructs of pathological inertia, mental bradyphrenia and disengagement and temporal re-ordering that underlie the dysexecutive phenotype associated with our model of ischaemic vascular disease (i.e., periventricular and deep white matter damage and related subcortical neuropathology associated with the basal ganglia, thalamus and pons), are considered together, we begin to see that this neuropathology may alter or derail how subcortical structures gate information back to the frontal lobes via disruptions of basal ganglia-thalamic pathways.

## 3. Theoretical and neuroanatomical considerations

When viewed as a whole, the clinical presentations outlined above suggest an ischaemic vascular disease in dementia characterized by periventricular and deep white matter alterations and/or related neuropathology involving the basal ganglia, thalamus and pons regardless of dementia diagnosis. Such ischaemic vascular disease appears to influence a dysexecutive syndrome involving underlying deficits of pathological inertia, bradyphrenia and disengagement and temporal re-ordering. These areas of dysfunction result in a pervasive executive impairment and involve an inability to 'gate' or filter information back to the frontal lobes. In determining possible neuroanatomical underpinnings of this dysexecutive phenotype several factors must be considered.

From a diagnostic point of view, the neuropathology underlying the ischaemic vascular disease of our various dementia groups appears to coalesce around periventricular and deep white matter alterations and/or related neuropathology involving the basal ganglia. Our patients with VaD by definition showed periventricular and deep white matter alterations and lacunar infarctions; however, upon closer inspection post-diagnosis, we found that the majority of lacunes were located within the centrum semiovale and various nuclei of the basal ganglia, thalamus and pons. The comorbid vascular risk factors investigated in our AD studies have been shown to cause structural alterations within periventricular and deep white matter in aging [36,37]. Furthermore, in addition to significant basal ganglia involvement seen in our dPD group, studies have found ischaemic vascular disease within periventricular and deep white matter regions [50]. All of these patient populations displayed some form of the dysexecutive syndrome and all have periventricular and deep white matter alterations and/or related neuropathology involving the basal ganglia. Lastly, when looking across dementia subtypes, we found that increased white matter hyperintensities quantified from FLAIR and T2-weighted MRI in periventricular and deep white matter regions increased the dysexecutive profile seen in dementia regardless of diagnosis. This suggests that the exact aetiology of ischaemic vascular disease resulting in periventricular and deep white matter alterations and/or related neuropathology involving the basal ganglia may not be as important as its anatomical distribution and severity.

From a theoretical point of view, first put forth by Luria [28] and later advanced by others [27,51], is the notion that certain aspects of executive functioning are related to specific, higher-level disorders of cognition while other aspects of behavior are more rudimentary and pervasive. It follows that the severity, as well as the specificity, of executive dysfunction is determined by whether a lesion actually resides in the frontal lobes and/or disrupts important connections between the frontal lobes and other areas of the brain. Ischaemic vascular disease and related subcortical neuropathology in dementia regardless of diagnosis involves periventricular and deep white matter that encompasses white matter tracts projecting from subcortical structures like the basal ganglia and thalamus into the prefrontal cortex [52]. These conclusions support the idea that ischaemic vascular disease characterized by white matter alterations and/or related neuropathology involving the basal ganglia regardless of dementia subtype while not directly involving the frontal lobes, negatively impacts executive functioning at a rather significant and pervasive level by means of its impact on important afferent and efferent neural connections involving the prefrontal cortex. This suggests that disruption of these prefrontal-basal ganglia-thalamic pathways [53] may explain the rather pervasive dysexecutive syndrome seen in these patients.

The role of the afferent and efferent connections between these subcortical structures and the prefrontal cortex, as well as the role of the basal ganglia and thalamus as major structures involved in cognition, should be explored as they relate to the dysexecutive syndrome outlined above. Across animal and human investigations, the notion of a gating mechanism responsible for the selection and subsequent production of appropriate responses has relied heavily on these structures and their intimate connections with the prefrontal cortex. Within the animal literature, derailment in the production of responses based on internal representation and inhibitory control are related to the prefrontal cortex [54] with resulting behaviors mediated through different regions of the caudate nucleus (see [55] for review). Across animal and human studies, the thalamus is thought to have a major regulatory role in the cortical synchronization of behavior via the basal ganglia (see [56] for review). Several researchers have speculated that the inhibitory actions of the basal ganglia serve as a selective gating mechanism that updates and directs operations that are then fed back to the frontal lobes through the thalamus [57,58]. Furthermore, neuroimaging studies suggest that automatic and over learned behavioral sequences are housed within the basal ganglia and thalamus [59]. When these structures are damaged, directly or indirectly (e.g., through white matter disconnectivity) the frontal lobes cannot effectively function to meet the needs that may be required when task demands change or become complex.

It is possible that the ischaemic vascular disease seen in our patients with dementia releases automatic behaviors making them more capable of interfering in cognition and resulting in the core conceptual deficits described as the dysexecutive phenotype within this review. That is, ischaemic vascular disease in our study participants appears to prohibit adequate engagement in task demands and when engaged, not only prohibits the behavior initiated to be preserved but also appears to prohibit the inhibition of irrelevant responses; in essence, decoupling the production of responses from the monitoring or gating of responses during and after task completion. Clearly, more directed neuroanatomical investigations need to be done to elucidate the notion that an impaired gating mechanism of the basal ganglia-thalamic pathway may be responsible for the dysexecutive syndrome associated with ischaemic vascular disease and related subcortical neuropathology in dementia before we can argue for a more neuroanatomically driven approach to this dysexecutive syndrome. However, we would advocate that ischaemic vascular disease regardless of dementia subtype may prove a surrogate for the role played by the basal ganglia and thalamus in monitoring the release of appropriate responses, thus weakening a subcortical gating mechanism.

## 4. Conclusion

We suggest that the dysexecutive syndrome associated with ischaemic vascular disease and related subcortical neuropathology in our dementia studies using a Boston Process Approach to executive functioning is caused by a derailment in three distinct but inter-related constructs (i.e., pathological inertia, bradyphrenia, disengagement and temporal re-ordering). As described above, this dysexecutive syndrome tends to be pervasive regardless of the task at hand or the modality used. More specifically, ischaemic vascular disease and related subcortical neuropathology in dementia regardless of subtype appears to prohibit adequate engagement in task demands and when engaged, prohibits the preservation of relevant behavior and the inhibition of irrelevant responses. This decoupling of response production from response monitoring is seen not only on executive tests but also when patients with ischaemic vascular disease are asked to copy the Rey Osterietth Complex Figure [60], to draw a clock to command and copy [61], and to retrieve information as assessed with a verbal serial list learning tasks [62]. In this sense, the pervasiveness of the dysexecutive syndrome associated with ischaemic vascular disease in dementia appears to intrude into other aspects of cognition.

We have put forth a hypothesis that elements of this dysexecutive syndrome may be caused by a derailed basal ganglia/ thalamic gating mechanism regardless of dementia subtype. The disruption of frontal lobe-basal ganglia-thalamic pathways secondary to increasing ischaemic vascular disease likely weakens gating mechanisms and ultimately may be the aetiology of both of the pervasiveness and poor regulation of executive control deficits [53,63]. New technologies such as advances in white matter tractography afforded by diffusion tensor imaging, as well as recently applied methods of magnetization transfer imaging and deep brain stimulation, may be able to provide additional information regarding how white matter connectivity (and disconnectivity) produces dysexecutive behavior in ischaemic vascular disease associated with dementia. Furthermore, studies using more traditional scoring methods across a variety of cognitive constructs in other dementia populations known to have ischaemic vascular disease characterized by periventricular and deep white matter alterations and/or related neuropathology involving the basal ganglia, thalamus and pons such as thalamic dementia or cerebral autosomal dominant arteriopathy with subcortical infarcts and leukoencephalopathy would assist in promoting the generalizability of our findings.

## Acknowledgment

We would like to acknowledgment the mentorship and guidance of Edith Kaplan, Ph.D. Without the insights provided by Dr. Kaplan none the research discussed in this paper would have been possible.
